# Mydriasis Stability During Cataract Surgery in Patients with Systemic Comorbidities Using a Standardised Combination of Intracameral Mydriatics and Anaesthetic

**DOI:** 10.3390/life15010119

**Published:** 2025-01-17

**Authors:** Joanna Dereń-Szumełda, Mariola Dorecka, Mirosław Dereń, Ewa Mrukwa-Kominek

**Affiliations:** 1 Ophthalmology Clinic, Military Institute of Aviation Medicine, 01-755 Warsaw, Poland; 2 University Clinical Centre named after Prof. K. Gibiński, Medical University of Silesia, 40-514 Katowice, Poland; marioladorecka@wp.pl (M.D.); emrowka@poczta.onet.pl (E.M.-K.); 3 Department of Psychophysiological Measurements and Human Factors Research, Research and Development Center, Military Institute of Aviation Medicine, 01-755 Warsaw, Poland

**Keywords:** mydriasis stability, cataract, mydriasis, diabetes, pseudoexfoliation syndrome

## Abstract

Background: This study aimed to evaluate mydriasis stability during cataract surgery in patients with systemic comorbidities such as diabetes mellitus (DM) and pseudoexfoliation syndrome (PXF) after a standardised combination of intracameral mydriatics and anaesthetic (SCIMA). Stable mydriasis is crucial for safe and effective phacoemulsification. Methods: Patients were included if they achieved pupil dilation ≥6.0 mm during the qualifying visit. A total of 103 patients were enrolled, divided into three groups: cataract with diabetes (C + DM group, *n* = 35), cataract with PXF (C + PXF group, *n* = 32), and cataract without those comorbidities (C group, *n* = 36). SCIMA was administered, and pupil diameters were measured at key surgical stages. Stability was defined as a pupil diameter of ≥6.0 mm without additional pharmacological intervention and no significant change in its diameter (≥3.0 mm). Results: Stable mydriasis was achieved in 90.3% of patients: 97.1% in the C + DM group, 90.6% in the C + PXF group, and 83.3% in the C group, with no statistically significant differences (*p* = 0.14). Conclusions: SCIMA effectively maintains mydriasis stability during cataract surgery, even in patients with systemic comorbidities, ensuring greater surgical safety.

## 1. Introduction

The global prevalence of cataract is growing as life expectancy continues to rise, particularly in developed nations. Cataract, characterised by the opacification of the lens, is primarily associated with ageing, but other factors such as diabetes mellitus (DM), UV exposure, and genetic predisposition also contribute to its development [1]. Of the 1 billion people whose vision impairment could have been prevented or has yet to be addressed, cataract is the leading cause of distance vision impairment or blindness, affecting 94 million individuals [2]. Although cataract is a treatable condition, the availability of surgical intervention is often limited in low- and middle- income countries, leading to disproportionately high rates of blindness in these regions [2,3]. In contrast, developed nations perform tens of thousands of cataract surgeries per million inhabitants each year, largely driven by advancements in surgical techniques such as phacoemulsification [4,5].

Cataract surgery is one of the most frequently performed procedures worldwide, with microincision phacoemulsification now the preferred method due to its reduced complication rates, shorter recovery times, and high success rates [4,6]. The phacoemulsification technique uses ultrasonic energy to emulsify the opaque lens, which is then aspirated from the eye and replaced with an artificial intraocular lens (IOL). However, the success of the procedure is heavily dependent on the ability to maintain adequate pupil dilation (mydriasis) throughout the surgery. Studies have shown that a pupil diameter of at least 6 mm is essential for optimal visualisation and safe manoeuvring during critical surgical steps such as capsulorhexis and IOL implantation [7,8]. Inadequate dilation increases the risk of intraoperative complications, including iris damage, posterior capsule rupture, and vitreous loss [9].

The standard approach to achieving mydriasis in cataract surgery involves the application of topical eye drops, typically a combination of phenylephrine and tropicamide, which induce pupil dilation by acting on the iris muscles. Phenylephrine stimulates α-adrenergic receptors in the dilator pupillae muscle, causing pupil dilation without affecting accommodation, while tropicamide blocks muscarinic receptors in the sphincter pupillae muscle, inhibiting cholinergic stimulation and leading to further dilation [4,10]. However, this method has several drawbacks. The bioavailability of topical agents is often low, with only a small percentage of the drug penetrating the eye, while the remainder is systemically absorbed, leading to potential cardiovascular side effects, particularly in elderly or at-risk patients [8,11,12]. Additionally, the onset of action is slow, requiring repeated administrations over the course of 20 to 30 min before surgery, which can be inconvenient for both patients and medical staff [9,13].

Furthermore, certain patient populations, such as those with diabetes mellitus (DM) or pseudoexfoliation syndrome (PXF), present additional challenges when it comes to achieving adequate mydriasis. DM is a well-established risk factor for impaired mydriasis due to autonomic neuropathy, which compromises the function of the iris dilator muscle [14]. Diabetic patients often present with smaller pupils, and the risk of intraoperative miosis is markedly higher in this group. Studies have shown that even when preoperative dilation is comparable to that of non-diabetic individuals, diabetic patients are prone to more pronounced intraoperative pupil constriction [7]. This can complicate critical surgical steps, including phacoemulsification and lens implantation, thereby increasing the risk of the complications mentioned above [9]. Given that up to 20% of cataract surgeries are performed on patients with diabetes, investigating the stability of mydriasis in this group is crucial to improving surgical outcomes [3,15].

Similarly, PXF poses unique challenges in cataract surgery. It is characterised by the accumulation of fibrillar material in the anterior chamber of the eye, leading to structural changes in the iris and zonules. These changes often result in poor preoperative pupil dilation and unpredictable intraoperative miosis, which can complicate the surgical process [16]. Furthermore, patients with PXF are at a higher risk of zonular instability, which exacerbates the difficulty of maintaining stable mydriasis throughout the procedure [17], further elevating the risk of intraoperative complications such as lens dislocation or vitreous prolapse [16,18].

In more complex cases, where unstable mydriasis occurs—marked by progressive pupil constriction during surgery—it becomes necessary to use mechanical devices such as pupil expanders or additional pharmacological interventions to maintain adequate dilation.

Given these limitations, there has been significant interest in alternative methods for achieving reliable and stable mydriasis during cataract surgery. An alternative for achieving mydriasis is the use of Mydriasert, Laboratoires Thea PHARMA S.A., Schaffhausen, Switzerland, an ophthalmic insert containing tropicamide and phenylephrine. This device is placed in the inferior conjunctival fornix, where it gradually releases its active ingredients, providing a more controlled and prolonged dilation compared to topical eye drops [11,19]. Mydriasert has been shown to be effective in achieving stable mydriasis with fewer systemic side effects due to its localised delivery mechanism [19]. However, despite its potential benefits, Mydriasert is currently unavailable in Poland, limiting its accessibility for cataract surgery patients in this region.

One of the most promising developments in recent years is the use of intracameral injections of mydriatic agents. Mydrane, a fixed-dose combination of tropicamide (0.02%), phenylephrine (0.31%), and lidocaine (1%), was introduced as a solution for achieving rapid and stable mydriasis through direct injection into the anterior chamber at the start of surgery [20,21,22]. The formulation provides the dual benefit of mydriasis and local anaesthesia, reducing the need for additional topical anaesthetic agents [23]. Lidocaine, a supplementary component in Mydrane, works by stabilising neuronal membranes, which prevents the conduction of nerve impulses, thereby providing effective anaesthesia during cataract surgery. In addition to its anaesthetic properties, lidocaine also supports pupil dilation, contributing to both patient comfort and procedural efficacy by maintaining stable mydriasis.

As mentioned before, this standardised combination of intracameral mydriatics and anaesthetic (SCIMA)—Mydrane, Laboratoires Thea PHARMA S.A., Schaffhausen, Switzerland, has been shown to induce rapid pupil dilation, with studies reporting that maximum dilation is achieved within 30–40 s after injection, significantly faster than the 20–30 min typically required for topical eye drops. In phase II [24] and phase III [25] clinical trials, Mydrane demonstrated non-inferior efficacy compared to standard topical regimens, with a high proportion of patients achieving and maintaining a pupil diameter of ≥6 mm throughout surgery [20,21,25]. Importantly, Mydrane’s effects are maintained for the duration of the procedure, eliminating the need for additional intraoperative mydriatics or mechanical dilation devices, which are often required in cases where topical drops are insufficient [3,8,10].

The use of SCIMA also offers significant advantages in terms of patient safety. The lower concentrations of active ingredients in this formulation reduce the risk of systemic side effects, such as hypertension and tachycardia, which can occur with higher doses of phenylephrine used in topical regimens [10,17]. This is particularly important for elderly patients or those with underlying cardiovascular conditions, who may be more susceptible to these adverse effects [8]. Furthermore, SCIMA’s rapid onset and consistent mydriatic effect optimises the surgical workflow, allowing for more efficient procedures and reduced preoperative waiting times [23].

However, despite these advantages, there is still a need for further research on the use of SCIMA in high-risk patient populations, such as those with DM and PXF. While SCIMA has shown efficacy in achieving and maintaining pupil dilation in the general population [20,21,24,25], its ability to provide stable mydriasis in patients with compromised iris function remains less well-documented. This is particularly relevant for patients with DM and PXF, two conditions frequently associated with smaller, less responsive pupils, which pose significant intraoperative challenges. Both conditions affect the dynamics of pupil dilation during cataract surgery, raising concerns about maintaining stable mydriasis throughout the procedure [3,8]. This unpredictability of pupil dynamics in these patient groups highlights the need to evaluate whether SCIMA can consistently maintain a pupil diameter of at least 6 mm, which is essential for preventing complications during critical phases of cataract surgery, such as capsulorhexis, phacoemulsification, and intraocular lens implantation.

By anticipating intraoperative pupil dynamics in high-risk populations and identifying mechanisms such as pre-existing iris damage, vascular abnormalities, and autonomic dysfunction that contribute to pupil constriction, it is possible to develop targeted interventions to prevent or mitigate pupil narrowing, ultimately improving the safety and efficacy of cataract surgery in patients with DM and PXF.

In conclusion, while SCIMA represents a promising tool for achieving rapid and stable mydriasis in the general cataract population, its role in managing high-risk patients with conditions such as DM and PXF requires further investigation. This study focuses on evaluating SCIMA’s ability to maintain stable mydriasis and prevent intraoperative miosis in these two high-risk groups. Intraoperative stability of mydriasis is essential for ensuring clear visualisation of the lens and smooth surgical manoeuvres, particularly in cases where the pupil size is compromised.

## 2. Methods

This research was observational and non-interventional, conducted in a real-world setting without randomization. It adhered to the ethical principles outlined in the Declaration of Helsinki. Prior to participation, all patients were fully informed about the study’s objectives and potential risks, and written consent was obtained. The study received approval from the Ethics Committee and was conducted between 2017 and 2019 at the University Clinical Centre, Medical University of Silesia, Katowice, Poland.

### 2.1. Study Design

The study involved measuring pupil diameter across two distinct visits. During the initial visit (V1), patients underwent an assessment to determine their eligibility for participation, in line with the guidelines outlined in the Summary of Product Characteristics. To qualify for the study, participants needed to achieve a pupil dilation of at least 6.0 mm, induced through the administration of mydriatic eye drops—specifically, 1% tropicamide and 10% phenylephrine. Measurements of the pupil diameter were taken no sooner than 30 min and no later than 40 min after the first application of these mydriatics, which were administered at approximately 10-min intervals for a total of three applications. This procedure was performed within a period ranging from one to several days before the scheduled cataract surgery. The measurements were taken using a NeurOptics VIP-200 pupilometer (SN 1203; NeurOptics, Irvine, CA, USA) to ensure maximum precision under non-sterile conditions.

To ensure the accuracy and reliability of the results, specific exclusion criteria were established to disqualify individuals whose medical conditions could potentially interfere with the study outcomes. Participants with both diabetes mellitus (DM) and pseudoexfoliation syndrome (PXF) were excluded due to the potential impact on pupil dynamics. Similarly, patients currently using α1-blockers were excluded, as these medications are known to influence pupil dilation. Further exclusion criteria included patients with any pre-existing iris damage, as well as those who required additional pharmacological agents or mechanical devices to achieve adequate pupil dilation. These criteria were designed to ensure an optimal response to the standard topical mydriatic protocol while still reflecting real-world clinical situations that may occur in everyday practice.

During this initial visit, detailed medical information was also collected from each participant. This included not only a review of their general medical history but also specific data relevant to ocular health and any treatments or medications that could potentially influence pupil reactivity. Gathering this information was crucial for accurately interpreting the obtained pupil measurements and ensuring a comprehensive understanding of the factors that might contribute to variability.

The second visit (V2) took place on the day of the cataract surgery. Measurements were conducted under sterile operating room conditions. Due to technical considerations and the requirement to maintain sterile conditions, the pupil diameter during surgery was measured using a sterile medical ruler, either paper-based or made of stainless steel, with an accuracy of 0.5 mm. In the medical centre where the study was performed, each patient routinely received local anaesthesia with 2% lidocaine gel prior to the cataract surgery. Once the patient was positioned on the operating table, pupil diameter measurements were taken at specific stages of the surgical procedure, including measurement:

V2-1: before the initial corneal incision and the administration of SCIMA

V2-2: 30–40 s after the administration of SCIMA into the anterior chamber

V2-3: just before capsulorhexis, following the administration of viscoelastic

V2-4: immediately before intraocular lens implantation

V2-5: just before the administration of cefuroxime and the conclusion of the surgery.

The maximum mydriasis was defined as the largest pupil diameter recorded throughout the entire phacoemulsification procedure.

A change in pupil diameter of less than 1.0 mm, as described in the Phase III study of Mydrane [25], was considered clinically insignificant, whereas a change of ≥3.0 mm was regarded as clinically significant. These definitions were adopted in this study as well.

The key stages of cataract surgery were identified as capsulorhexis, lens mass phacoemulsification, and intraocular lens implantation. Stability of pharmacological mydriasis was defined as being achieved when the pupil diameter during measurements V2-3 and V2-4 at key stages of cataract surgery remained equal to or greater than 6.0 mm without the need for additional dilating agents, and when no clinically significant change in pupil diameter (≥3.0 mm) was observed between these measurements during the key surgical stages.

This approach ensured a standardised method for evaluating the response to the mydriatic protocol and provided reliable data regarding the stability of pupil dilation throughout the surgical procedure.

### 2.2. Study Participants

A total of 103 patients (103 eyes) were included in the final analysis, with a mean age of 71.7 years (±7.45). Of these, 85 patients (82.5%) were women, and 82 patients (79.6%) had a history of hypertension. Patients were categorised into three groups: those with cataract and PXF (C + PXF; *n* = 32), cataract and DM (C + DM; *n* = 35), and a control group with cataract but no PXF or DM (C; *n* = 36).

Among the patients with diabetes mellitus (DM), 20 (57.1%) were on oral medications only, 15 (42.9%) were receiving insulin therapy, and 6 (17.1%) had diabetic retinopathy.

Throughout the procedure, surgeons were ethically permitted to implement additional measures, such as adrenaline or iris retractors, to ensure patient safety and maintain proper pupil dilation when necessary. Any deviations from the standard protocol were meticulously documented. To standardise the study, the same type of cohesive viscoelastic was used for all patients during phacoemulsification. The study protocol required as well that any use of a different type or additional dose of viscoelastic outside the established procedure be documented. For statistical analysis, only pupil diameter measurements taken prior to any additional interventions were included, ensuring that the dilation achieved was solely attributed to SCIMA.

### 2.3. Statistical Methods

A two-tailed *p*-value of less than 0.05 was considered indicative of statistical significance. All calculations were carried out using Statistica (Data Analysis Software System), version 13.3.0 (TIBCO Software Inc., Redwood City, CA, USA, 2017) and Set Plus, version 3.0.67 (StatSoft Polska Sp. z o.o., Kraków, Poland).

Continuous variables are presented as either mean (±standard deviation) or median with interquartile range (Q1–Q3), depending on the nature of the data distribution. Additionally, the minimum and maximum values of continuous variables are provided to indicate the full range. Selected graphs include 95% confidence intervals for the means. Categorical variables are represented by the number of observations and corresponding percentages. To assess the normality of the distribution of continuous variables, both a visual inspection and the Shapiro–Wilk test were employed.

For determining the significance of differences between means, either Student’s *t*-test or the Mann–Whitney U test was applied, based on the distribution of the data. When comparing continuous variables across more than two groups, either ANOVA or the Kruskal–Wallis H test was used, depending on whether the data followed a normal distribution. For repeated measures across different visits, repeated-measures ANOVA was utilised. In post hoc analysis for continuous variables, Tukey’s honestly significant difference test was applied to identify specific group differences.

For categorical data, the χ2 (chi-square) test was used to assess significance, with Yates’s correction applied if any expected frequency was less than 5. In cases involving multiple comparisons of categorical data, the classic Bonferroni correction was employed, adjusting the significance level by multiplying the *p*-value by the number of comparisons conducted.

This approach ensured robust statistical evaluation, taking into account both the nature of the data and the specific hypotheses being tested.

## 3. Results

### 3.1. Stability of Mydriasis

**Figure 1:**
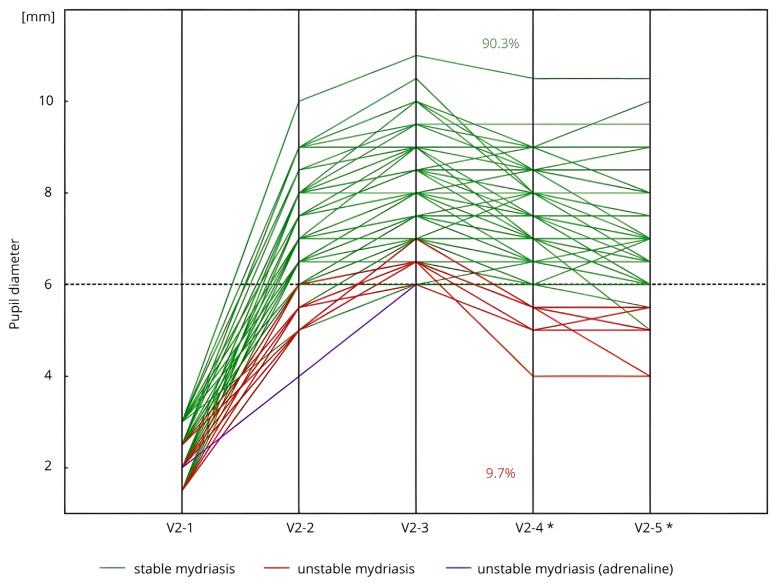
Stability of pharmacological mydriasis (measurements V2-3–V2-4) in all examined patients (*n* = 103) during the Second Visit (V2). * *n* = 102, due to the administration of an adrenaline injection before the V2-4 measurement in 1 patient. Measurement: V2-1: before the initial corneal incision and the administration of SCIMA; V2-2: 30–40 s after the administration of SCIMA into the anterior chamber; V2-3: just before capsulorhexis, following the administration of viscoelastic; V2-4: immediately before intraocular lens implantation; V2-5: just before the administration of cefuroxime and the conclusion of the surgery.

Mydriasis was stable in 90.3% of all study participants (*n* = 93/103, Figure 1). In Group C, stability was observed in 83.3% of patients (*n* = 30/36), whereas in Group C + DM, the rate was 97.1% (*n* = 34/35), and in Group C + PXF, stable mydriasis was noted in 90.6% of patients (*n* = 29/32). In these patients, the pupil diameter remained ≥6.0 mm during the key stages of cataract surgery without the need for additional dilating agents, and no clinically significant decrease in pupil diameter (≥3.0 mm) was noted.

Among the patients with unstable mydriasis, one individual (from Group C) required an additional pupil-dilating agent (adrenaline injection) just before intraocular lens implantation (measurement V2-4). For the remaining nine patients, the pupil diameter at V2-4 was below 6.0 mm.

### 3.2. Pupil Diameter

At each of the key stages of cataract surgery (measurements V2-3 to V2-4), 91.2% of all patients (*n* = 93/102), among those who did not require additional pupil-dilating agents, had a pupil diameter of ≥6.0 mm. Furthermore, after achieving maximum mydriasis, 89.2% of patients (*n* = 91/102) maintained a pupil diameter of ≥6.0 mm until the end of the surgery (measurement V2-5) without the use of additional dilating agents.

**Figure 2:**
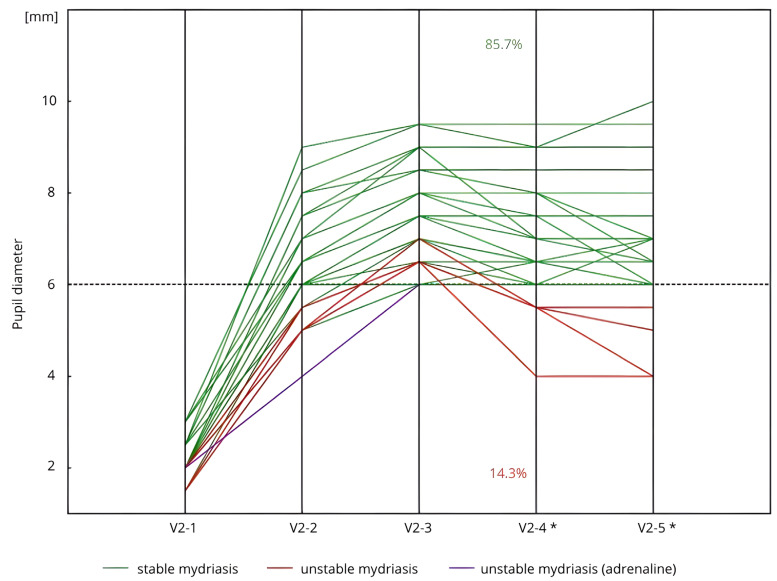
Stability of pharmacological mydriasis (measurements V2-3–V2-4) in patients with cataract (Group C) but without diabetes and without PXF (*n* = 36) during the Second Visit (V2). * *n* = 35, due to the administration of an adrenaline injection before the V2-4 measurement in 1 patient. Measurement: V2-1: before the initial corneal incision and the administration of SCIMA; V2-2: 30–40 s after the administration of SCIMA into the anterior chamber; V2-3: just before capsulorhexis, following the administration of viscoelastic; V2-4: immediately before intraocular lens implantation; V2-5: just before the administration of cefuroxime and the conclusion of the surgery.

In Group C, at each key stage of cataract surgery, 85.7% of patients (*n* = 30/35), among those who did not receive additional pupil-dilating agents, maintained a pupil diameter of ≥6.0 mm. The same proportion of patients retained a pupil diameter of ≥6.0 mm from achieving maximum mydriasis until V2-5 without the use of any additional dilating agents (Figure 2).

**Figure 3:**
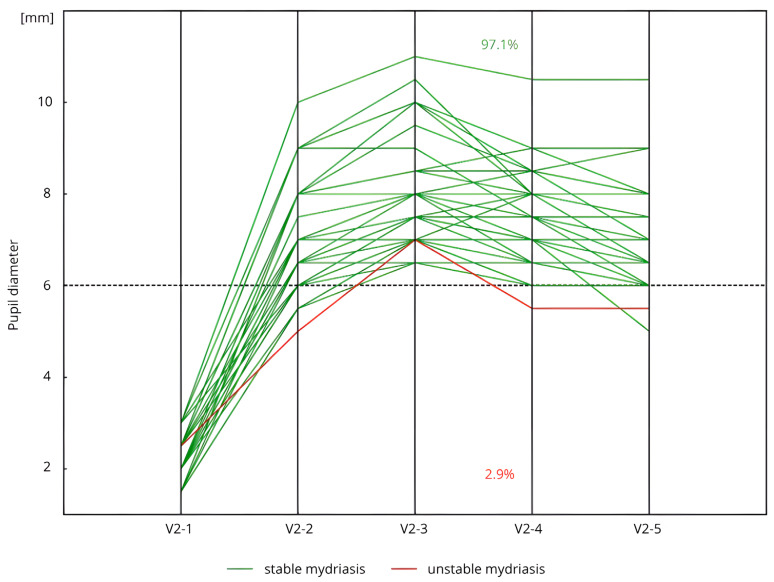
Stability of pharmacological mydriasis (measurements V2-3–V2-4) in patients with cataract and DM (Group C + DM) (*n* = 35) during the Second Visit (V2). Measurement: V2-1: before the initial corneal incision and the administration of SCIMA; V2-2: 30–40 s after the administration of SCIMA into the anterior chamber; V2-3: just before capsulorhexis, following the administration of viscoelastic; V2-4: immediately before intraocular lens implantation; V2-5: just before the administration of cefuroxime and the conclusion of the surgery.

In Group C + DM, 97.1% of patients (*n* = 34/35), at each key stage of cataract surgery, maintained a pupil diameter of ≥6.0 mm without the need for additional dilating agents. Additionally, after achieving maximum mydriasis, 94.3% of these patients (*n* = 33/35) maintained a pupil diameter of ≥6.0 mm at each stage of cataract surgery without the use of supplementary pupil-dilating agents (Figure 3).

**Figure 4:**
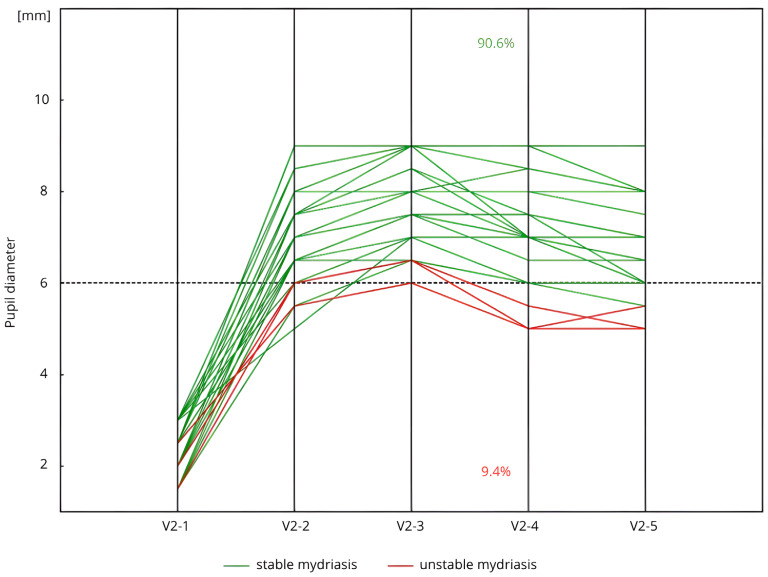
Stability of pharmacological mydriasis (measurements V2-3–V2-4) in patients with cataract and PXF (Group C + PXF) (*n* = 32) during the second visit (V2). Measurement: V2-1: before the initial corneal incision and the administration of SCIMA; V2-2: 30–40 s after the administration of SCIMA into the anterior chamber; V2-3: just before capsulorhexis, following the administration of viscoelastic; V2-4: immediately before intraocular lens implantation; V2-5: just before the administration of cefuroxime and the conclusion of the surgery.

In Group C + PXF, 90.6% of patients (*n* = 29/32), at each key stage of cataract surgery, maintained a pupil diameter of ≥6.0 mm without any additional pharmacological support. Moreover, 87.5% of these patients (*n* = 28/32) continued to have a pupil diameter of ≥6.0 mm until V2-5 without the need for further pupil dilation (Figure 4).

The differences between the groups (C vs. C + DM vs. C + PXF) in the number of patients maintaining a pupil diameter of ≥6.0 mm at each stage of the cataract surgery, without requiring additional dilating agents, from measurements V2-3 to V2-5, were not statistically significant (*p* = 0.48).

In nine individuals at one of the key stages of cataract surgery, the pupil diameter decreased below 6.0 mm, but no additional pharmacological support was used in these patients.

A comparison of the mean pupil diameter between the groups: Group C, Group C + DM, and in Group C + PXF during the V2 is presented in Table 1.

### 3.3. Pupil Diameter Change

The average change in pupil diameter from measurement V2-3 to V2-4 (*p* = 0.85), as well as from measurement V2-3 to V2-5 (*p* = 0.35), did not differ significantly between the groups (C vs. C + DM vs. C + PXF; Table 1). The change in average pupil diameter between measurements V2-3 and V2-4 was −0.5 mm in both Group C + DM (*n* = 35) and Group C (*n* = 35), while in Group C + PXF (*n* = 32), it was 0.4 mm. The decrease in average pupil diameter between measurements V2-3 and V2-5 was as follows: in Group C + DM (*n* = 35), it decreased by 0.9 mm; in Group C + PXF (*n* = 32), by 0.8 mm; and in Group C (*n* = 32), by 0.6 mm.

### 3.4. Pupil Diameter Change After Achieving Maximum Mydriasis

**Figure 5:**
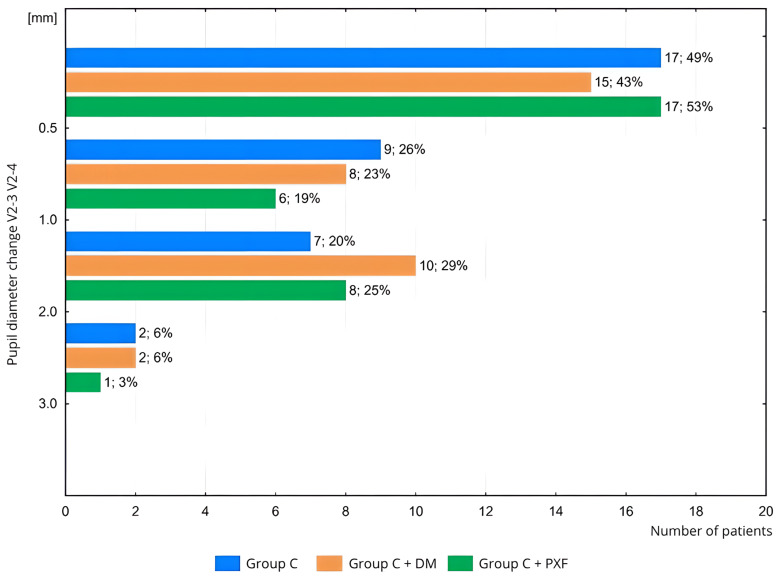
Comparison of pupil diameter changes between the groups: with cataract, with cataract and diabetes, and with cataract and PXF at key stages of cataract phacoemulsification during the second visit. Next to the bars, the following are provided: the number of patients; the percentage of patients in the group. Measurement: V2-3: just before capsulorhexis, following the administration of viscoelastic; V2-4: immediately before intraocular lens implantation. Groups: C—with cataract, C + DM—with cataract and DM; C + PXF—with cataract and PXF.

At key stages of the phacoemulsification procedure (measurements V2-3 to V2-4; Figure 5), a clinically insignificant change in pupil diameter (<1.0 mm) was observed in 75% of patients in Group C (*n* = 26/35), 66% of patients in Group C + DM (*n* = 23/35), and 72% of patients in Group C + PXF (*n* = 23/32). The differences between the groups were not statistically significant (*p* = 0.72). No patient experienced a clinically significant change in pupil diameter (≥3.0 mm) during the key stages of the phacoemulsification procedure.

**Figure 6:**
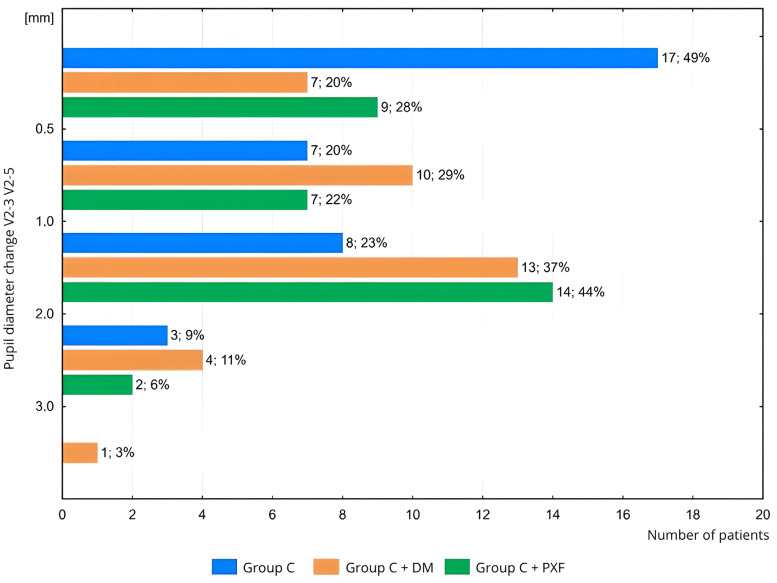
Comparison of pupil diameter changes between the groups: with cataract, with cataract and diabetes, and with cataract and PXF from the capsulorhexis stage to the end of the cataract surgery during the Second Visit. Next to the bars, the following are provided: the number of patients; the percentage of patients in the group. Measurement: V2-3: just before capsulorhexis, following the administration of viscoelastic; V2-5: just before the administration of cefuroxime and the conclusion of the surgery. Groups: C—with cataract, C + DM—with cataract and DM; C + PXF—with cataract and PXF.

In the time interval between just before capsulorhexis (following viscoelastic administration) and the administration of cefuroxime, right before the end of the phacoemulsification procedure (measurements V2-3 to V2-5; Figure 6), a clinically insignificant change in pupil diameter (<1 mm) was noted in 69% of patients in Group C (*n* = 24/35), 49% of patients in Group C + DM (*n* = 17/35), and 50% of patients in Group C + PXF (*n* = 16/32). The differences between the groups were not statistically significant (*p* = 0.17).

A clinically significant change in pupil diameter (≥3.0 mm) between measurements V2-3 and V2-5 was observed in only 3% of patients (*n* = 1/35) in Group C + DM (Figure 6). The differences between the study groups were also not statistically significant (*p* = 0.38).

### 3.5. Adverse Events

During V1, the application of mydriatic drops caused ocular discomfort described by patients as “stinging” and “burning.” No other adverse reactions to the standard topical mydriatics were observed in the study group.

During V2, no ocular or systemic adverse effects from SCIMA were reported. Only one patient in Group C + PXF experienced moderate intraoperative floppy iris syndrome (IFIS). Additionally, in a different instance, anterior chamber shallowing was noted. In one patient, despite the absence of evident risk factors noted during V1 for intraoperative complications, additional pupil-dilating agents were required due to unstable mydriasis (details below).

### 3.6. Use of Additional Pupil-Dilating Agents

Only one patient required additional pupil-dilating agents during cataract surgery. Due to an unstable and narrowing pupil before IOL implantation, adrenaline (0.167 mg/mL, 1:6000 dilution) was injected into the anterior chamber between measurements V2-3 and V2-4. As a result, subsequent pupil measurements from V2-4 onwards were excluded from statistical analysis, and pupil stability was considered unachieved. The patient was female and had no PXF or diabetes.

### 3.7. Surgery Duration Time

The average surgery time was significantly shorter (*p* = 0.02) in the C + DM group (14.6 min) compared to the C group (17.0 min). According to the methodology of the study, the patient from the C group, after the adrenaline injection but before the V2-4 measurement, was not included in these calculations. The surgery time for this patient was 15 min. The average surgery time in the C + PXF group was not statistically significantly different (16.9 min) from the other groups (*p* = 1.0; *p* = 0.14, respectively).

## 4. Discussion

The aim of this study was to evaluate the stability of mydriasis following the use of SCIMA during key stages of phacoemulsification surgery and compare the results across three patient groups: those with cataract and diabetes (Group C + DM), cataract and pseudoexfoliation syndrome (Group C + PXF), and cataract without these comorbidities (Group C).

Achieving and maintaining sufficient pupil dilation throughout cataract surgery, particularly during its key stages, ensures both safety and optimal working conditions for the surgeon. Phacoemulsification, by mechanical manipulation of the iris during surgery, not only may lead to the damage of the iris dilator muscle, but also triggers synthesis of prostaglandins in ocular tissues, causing reactions such as pupil constriction, conjunctival hyperaemia, disruption of the blood-aqueous barrier and increased intraocular pressure [26,27]. The more the iris is manipulated—due to prolonged surgery or narrow pupil—the more prostaglandins are released, increasing the risk of miosis. This risk is notably higher in patients with diabetes [28] and PXF [29]. Mydriatics can be washed out during surgery by the natural flow of aqueous humour and irrigation fluids, further contributing to pupil constriction. Moreover, inadequate adrenergic stimulation has been identified as a key factor in intraoperative pupil constriction [30]. As a consequence, reduced mydriatic effect, combined with reflexive pupil constriction during surgery, heightens the risk of intraoperative complications. A hypothesis has emerged suggesting that fully saturating the iris receptors with mydriatic agents administered directly into the anterior chamber could result in more stable mydriasis throughout the procedure.

The key stages of phacoemulsification are fundamental steps in cataract surgery and should be performed under the surgeon’s direct visual control. Stable mydriasis ensures this visibility and the safe performance of the procedure. Therefore, this study focused on assessing mydriasis stability during these key surgical stages.

In a 2013 European Observatory of Cataract Surgery Survey [31], surgeons ranked stable mydriasis and maximum pupil dilation as the most important components of mydriasis.

In a pioneering study on SCIMA from 2016 by Labetoulle et al. [25], in contrast to the present study, patients with systemic conditions such as PXF or those undergoing insulin treatment for diabetes were excluded. When comparing average pupil diameters at key stages of phacoemulsification, Labetoulle et al. [25] reported stable mydriasis, with pupil diameters of 7.67 mm before capsulorhexis and 7.71 mm before lens implantation. The more stringent patient selection in that study likely contributed to the higher stability of mydriasis, whereas in the present study, this parameter was slightly lower. The average pupil diameter was 7.4 mm before capsulorhexis (measurement V2-3) and decreased to 6.9 mm just before lens implantation (measurement V2-4) in the Group C. It should be noted that in the aforementioned study by Labetoulle et al. [25], patients included had achieved a pupil diameter of ≥7.0 mm following the administration of standard topical mydriatic eye drops, unlike the present study, where a threshold of ≥6.0 mm was used, which may also explain the differences observed between the studies. Furthermore, in the presented study, in accordance with the Summary of Product Characteristics [20], only a single dose of 0.2 mL of SCIMA was administered. In the study by Labetoulle et al. [25], 26.5% of patients, in addition to the standard initial dose of 0.2 mL administered at the beginning of the procedure, received more than one injection (0.1 mL) of SCIMA into the anterior chamber [24,32].

Multiple studies have demonstrated that diabetic patients tend to have smaller pupils and a reduced response to mydriatic agents [33,34]. This diminished mydriasis is likely linked to disrupted iris innervation and a loss of its mechanical properties [35]. Consequently, individuals with diabetes are at greater risk of a weaker reaction to pupil-dilating drugs compared to healthy individuals [36]. Autonomic nerve damage in diabetes can affect both the sympathetic (dilator pupillae) and parasympathetic (sphincter pupillae) pathways [37,38]. Additionally, long-term diabetes may cause structural changes in the iris, including connective tissue degeneration, vascular damage, and the accumulation of glycogen and lipids, reducing its elasticity [39,40,41].

In a subsequent study by Labetoulle et al. [21], the average pupil diameter was compared in patients with previously demonstrated stable mydriasis after SCIMA administration [25]: in patients with non-insulin-treated DM and patients without DM. In the latter group, the average pupil diameter was 7.7 mm and remained unchanged at key stages of the surgery. Meanwhile, in the group of patients with non-insulin-dependent diabetes, the average pupil diameter was 7.4 mm just before capsulorhexis and 7.5 mm before lens implantation, which was also considered stable. Compared to the results of the Labetoulle et al. [21] study, the average pupil diameter in the C + DM group in the present study was larger before capsulorhexis at 8.0 mm, decreasing to 7.5 mm in measurement V2-4. Clinically, this change was not significant, and, similar to the findings of Labetoulle et al. [21], mydriasis could be considered stable. The clinically insignificant difference likely resulted from the previously mentioned inclusion criteria in the Labetoulle et al. [21] study (pupil diameter ≥7.0 mm) and the use of additional drug doses (more than one injection of SCIMA).

In ophthalmology, PXF is associated with a predisposition to poor mydriasis, the formation of posterior synechiae, zonular instability predisposing to lens dislocation, and glaucoma. The loss of elasticity in connective tissue and the atrophy of iris muscles due to hypoxia result in a diminished capacity for iris dilation. These pathological changes provide insight into the range of complications that may occur during intraocular procedures in patients with PXF. Knowledge of the pathophysiology of PXF and its practical implications for cataract surgery enables the prevention and mitigation of surgical complications through the careful application of pharmacological and mechanical prophylactic measures [42,43,44,45].

Following an analysis of the available literature, a lack of information was identified regarding the efficacy of mydriasis after the administration of SCIMA in a specific group of patients with PXF. It is particularly notable that in phase II and III trials [24,25], this condition was listed as an exclusion criterion. Nuzzi et al. [35] observed reduced efficacy of mydriasis in 76.2% of patients within a group that included individuals with PXF. However, they did not specify the proportion of patients with PXF or the percentage of other conditions that might have influenced the effect of mydriasis. There are only a few reports in the literature examining changes in pupil diameter and the extent of pharmacological mydriasis in eyes affected by PXF. Yiğit et al. [46] and Tekin et al. [47] addressed this topic, confirming that PXF causes structural abnormalities in the iris, which affect both pupil size and the achieved mydriasis.

In the present study, the change in pupil diameter between measurements V2-3 and V2-4 across all groups was comparable and did not differ significantly. The average reduction in pupil size ranged between 0.4 and 0.5 mm. According to this study’s criteria, this change (<1.0 mm) was not clinically significant in most patients: 75% (*n* = 26/35) in the C group, 66% (*n* = 23/35) in the C + DM group, and 72% (*n* = 23/32) in the C + PXF group. None of the patients experienced a clinically significant reduction in pupil diameter (≥3.0 mm) during the key stages of phacoemulsification (V2-3 to V2-4).

According to Chiambaretta et al. [24], the stability of mydriasis was greater when a combination of intracameral drugs was used compared to topically administered drops. The average change in pupil diameter (from capsulorhexis to the end of surgery) in the group receiving intracameral mydriatics was −0.22 mm, while in the group using standard topical mydriatics, it was −1.67 mm. The authors explained this by noting that after reaching 95% of maximum mydriasis within an average of 28.6 s, the pupil continued to slowly dilate [24,48].

Although in the present study, the change in pupil diameter at key stages of phacoemulsification was the main measurement, for comparison with other studies, the change in pupil diameter between measurements V2-3 and V2-5 (from just before capsulorhexis to the end of surgery) was also assessed. These differences were not statistically significant between the reference group and the groups with systemic conditions, namely DM and PXF. In the C group, 69% of patients (*n* = 24/35) experienced a clinically insignificant change in pupil diameter of <1.0 mm, compared to 50% (*n* = 16/32) in the C + PXF group and 49% (*n* = 17/35) in the C + DM group. A clinically significant change in pupil diameter (≥3.0 mm) between key stages of cataract surgery (V2-3 to V2-5) occurred only in 3% of patients (*n* = 1/35) in the C + DM group. No clinically significant changes in pupil diameter ≥3.0 mm were observed in either the C or C + PXF group.

It this study, pupil constriction of <1.0 mm between measurements V2-3 and V2-5 occurred in more patients compared to the V2-3 to V2-4 interval, which aligns with typical clinical observations where the pupil size in V2-5 is generally smaller than in V2-4. The change in pupil diameter between V2-3 and V2-5 was slightly larger in the C + DM group than in the C group, which could support the conclusion that miosis (pupil constriction) is a common ocular symptom in diabetes [49]. However, this difference did not reach statistical significance. According to the authors, the greater change in pupil diameter could be attributed to multiple factors, including the removal of viscoelastic material, which stabilises the iris and has an additional mydriatic effect, as well as the release of prostaglandins triggered by irritation of the iris caused by the implanted lens and the irrigation and aspiration process. Once the lens is implanted and the viscoelastic material is washed out from the eye, the pupil diameter no longer has a significant impact on the final outcome or the safety of the phacoemulsification. Therefore, maintaining large mydriasis at this stage is not obligatory.

The average change in pupil diameter in the presented study (measurements V2-3 to V2-5) for the C group was −0.6 mm, which was larger than the −0.22 mm reported in the Phase III trial analysed by Chiambaretta et al. [24]. Additionally, in the Phase III trial [24,25], a greater number of patients (89.3%; *n* = 208/233) compared to the C group in this study (69%; *n* = 24/35) experienced a clinically insignificant change in pupil diameter of <1.0 mm. None of the patients in the C group experienced a clinically significant change in pupil diameter of ≥3.0 mm during measurements V2-3 to V2-5. The differences in measurement results between the groups could be explained also by the different inclusion criteria used in both studies.

Similarly, a more recent study by Labetoulle et al. [21] used the same inclusion criteria as the Phase III trial, and the average change in pupil diameter after SCIMA administration (from capsulorhexis to the end of surgery) in the cataract and diabetes group was −0.11 mm, compared to −0.24 mm in the cataract-only group. This difference was at least half the size of the changes observed in the C + DM and C groups in the present study, where the average changes in pupil diameter (V2-3 to V2-5) were −0.9 mm and −0.6 mm, respectively. Similarly to Chiambaretta et al. [24], Labetoulle et al. [21] found a higher proportion of patients without a clinically significant change in pupil diameter than in the present study. In the cataract and diabetes group from the Labetoulle et al. [21] study, 82.6% of patients (*n* = 19/23) had no clinically significant change in pupil diameter of <1.0 mm, compared to 90.0% (*n* = 189/210) in the reference group (without diabetes). In the current study, a clinically insignificant change in pupil diameter of <1.0 mm (V2-3 to V2-5) occurred in 49% (*n* = 17/35) of patients in the C + DM group and 69% (*n* = 24/35) of patients in the C group. This suggests that patients who achieved a pupil diameter of ≥7.0 mm during qualification after standard topical mydriatics experienced smaller changes in pupil diameter than patients who had a pupil diameter of ≥6.0 mm during qualification. In the present study, the average pupil diameter at the end of cataract surgery did not differ significantly between the C, C + DM, and C + PXF groups.

The authors fully acknowledge the limitations of the sample size, even though it was determined in accordance with relevant statistical requirements. However, since this comparison has not been previously undertaken, it provides a new perspective and opens the possibility of expanding the study population in future research.

Despite minor differences of tenths of a millimetre, the results of this study are consistent with those obtained in other studies, indicating the stable nature of mydriasis following the use of SCIMA, even in patients with comorbidities such as diabetes mellitus and pseudoexfoliation syndrome. Furthermore, regardless of the presence of those conditions that negatively affect mydriasis, the achieved pupil dilation during the key stages of phacoemulsification was classified as large [14], as the average pupil diameter in the C + DM and C + PXF groups ranged between 6.0 and 8.0 mm. The authors concluded that the use of SCIMA in the C + DM and C + PXF groups ensured both stable and optimal mydriasis during the key stages of phacoemulsification.

## 5. Conclusions

Maintaining stable mydriasis in patients with cataract and systemic conditions, such as diabetes and PXF, during the key stages of cataract phacoemulsification is a crucial factor ensuring the safety of the procedure. The use of SCIMA in these patients allows for the preservation of stable mydriasis.

## Figures and Tables

**Table 1:** Comparison of mean pupil diameter between the groups: with cataract, with cataract and diabetes, and with cataract and PXF during the second visit.

Second Visit		Pupil Diameter [mm]						
		(A) Group C (*n* = 36)	(B) Group C + DM (*n* = 35)	(C) Group C + PXF (*n* = 32)	*p*	Post Hoc Tests		
						*p* A vs. B	*p* A vs. C	*p* B vs. C
V2-3	before capsulorhexis	7.4 (±1.03)	8.0 (±1.18)	7.8 (±0.89)	0.04	0.04	0.30	0.64
V2-4	before intraocular lens implantation	6.9 (±1.21) *	7.5 (±1.04)	7.3 (±1.13)	0.14	-	-	-
V2-5	before the end of surgery	6.8 (±1.35) *	7.1 (±1.14)	7.0 (±1.16)	0.56	-	-	-
Pupil diameter change V2-4–V2-3		−0.5 (±0.63) *	−0.5 (±0.76)	−0.4 (±0.58)	0.85	-	-	-
Pupil diameter change V2-5–V2-3		−0.6 (±0.82) *	−0.9 (±0.83)	−0.8 (±0.68)	0.35	-	-	-

Values are presented as mean (± standard deviation); If *p* (probability) was not significant, post-hoc tests were not performed, and this is indicated by a hyphen (“-”); * *n* = 35, due to the administration of an adrenaline injection before the V2-4 measurement in 1 patient; Groups: C—with cataract, C + DM—with cataract and DM; C + PXF—with cataract and PXF.

## Data Availability

Data are available from the corresponding author upon reasonable request.
